# *Aedes aegypti* Shows Increased Susceptibility to Zika Virus via Both In Vitro and In Vivo Models of Type II Diabetes

**DOI:** 10.3390/v14040665

**Published:** 2022-03-23

**Authors:** Sasha R. Azar, Rafael K. Campos, Ruimei Yun, Taylor Strange, Shannan L. Rossi, Kathryn A. Hanley, Nikos Vasilakis, Scott C. Weaver

**Affiliations:** 1 Department of Pathology, The University of Texas Medical Branch, Galveston, TX 77555-0609, USA; srazar@utmb.edu (S.R.A.); rkkroonc@utmb.edu (R.K.C.); tastrang@utmb.edu (T.S.); slrossi@utmb.edu (S.L.R.); 2 Department of Microbiology and Immunology, The University of Texas Medical Branch, Galveston, TX 77555-0610, USA; ruyun@utmb.edu; 3 Center for Biodefense and Emerging Infectious Diseases, University of Texas Medical Branch, Galveston, TX 77555-0609, USA; 4 Center for Tropical Diseases, University of Texas Medical Branch, Galveston, TX 77555-0609, USA; 5 Institute for Human Infection and Immunity, University of Texas Medical Branch, Galveston, TX 77555-0610, USA; 6 Department of Biology, New Mexico State University, Las Cruces, NM 88003, USA; khanley@nmsu.edu; 7 World Reference Center for Emerging Viruses and Arboviruses, University of Texas Medical Branch, Galveston, TX 77555-0609, USA; 8 Center for Vector-Borne and Zoonotic Diseases, University of Texas Medical Branch, Galveston, TX 77555-0609, USA; 9 Department of Preventive Medicine and Population Health, The University of Texas Medical Branch, Galveston, TX 77555, USA

**Keywords:** *Aedes aegypti*, zika virus, type II diabetes, TGF-β, immunological cross-talk

## Abstract

Chronic conditions like type II diabetes (T2DM) have long been known to exacerbate many infectious diseases. For many arboviruses, including Zika virus (ZIKV), severe outcomes, morbidity and mortality usually only occur in patients with such pre-existing conditions. However, the effects of T2DM and other pre-existing conditions on human blood (e.g., hypo/hyperinsulinemia, hyperglycemia and hyperlipidemia) that may impact infectivity of arboviruses for vectors is largely unexplored. We investigated whether the susceptibility of Aedes aegypti mosquitoes was affected when the mosquitoes fed on “diabetic” bloodmeals, such as bloodmeals composed of artificially glycosylated erythrocytes or those from viremic, diabetic mice (LEPRDB/DB). Increasing glycosylation of erythrocytes from hemoglobin A1c (HgbA1c) values of 5.5–5.9 to 6.2 increased the infection rate of a Galveston, Texas strain of Ae. aegypti to ZIKV strain PRVABC59 at a bloodmeal titer of 4.14 log10 FFU/mL from 0.0 to 40.9 and 42.9%, respectively. ZIKV was present in the blood of viremic LEPRDB/DB mice at similar levels as isogenic control C57BL/6J mice (3.3 log10 FFU/mL and 3.6 log10 FFU/mL, respectively. When mice sustained a higher ZIKV viremia of 4.6 log10 FFU/mL, LEPRDB/DB mice infected 36.3% of mosquitoes while control C57BL/6J mice with a viremia of 4.2 log10 FFU/mL infected only 4.1%. Additionally, when highly susceptible Ae. aegypti Rockefeller mosquitoes fed on homozygous LEPRDB/DB, heterozygous LEPRWT/DB, and control C57BL/6J mice with viremias of ≈ 4 log10 FFU/mL, 54%, 15%, and 33% were infected, respectively. In total, these data suggest that the prevalence of T2DM in a population may have a significant impact on ZIKV transmission and indicates the need for further investigation of the impacts of pre-existing metabolic conditions on arbovirus transmission.

## 1. Introduction

The last century has seen a marked rise in both the emergence and incidence of arthropod-borne virus (arbovirus) infections [1,2,3,4] and the prevalence of chronic medical conditions [5,6,7,8] in the human population. On the arbovirus front, some long-recognized threats, such as the dengue and yellow fever viruses, have surged in recent decades [3,9,10,11,12], while others, most prominently the Zika (ZIKV) and chikungunya viruses (CHIKV), have emerged into global prominence only within the last ten years [9,10,11,12]. One in three adult patients suffers from multiple chronic medical conditions simultaneously, a number that is anticipated to double in developed countries by 2035 [5]. One analysis of data from the National Health Interview Survey in 2012 found that approximately 117 million US adults (≈50%) had at least one of ten chronic conditions (hypertension, coronary heart disease, stroke, diabetes, cancer, arthritis, hepatitis, weak or failing kidneys, current asthma, or chronic obstructive pulmonary disease), while about one in four suffered from two or more chronic conditions [8]. As many of the chronic conditions listed above are associated with hematologic changes (local and systemic alterations of blood pH, hyper- and hypoinsulinemia, hyperglycemia, and an altered basal circulation of inflammatory and anti-inflammatory cytokines), it is reasonable to hypothesize that they might impact the progression of arboviral replication, disease and transmission. The observation that infection with many arboviruses [13,14,15,16,17,18,19,20,21] results in more severe disease outcomes and mortality in patients with such pre-existing conditions supports this hypothesis. 

In the current study, we focused on Type II diabetes mellitus (T2DM), which causes hematologic manifestations including hyperglycemia, hyperinsulinemia (and eventual hypoinsulinemia), metabolic acidosis and hyperlipidemia, as well as chronic inflammation [22]. Jean-Baptiste et al. reported that the clinical manifestations of CHIKV infection are more prevalent and longer-lasting in diabetic than non-diabetic patients [23]. However, the documented hematological effects of T2DM on the arbovirus infection of mosquito vectors have not been explored. Indeed, comparatively little attention has been afforded to the impact of blood-borne factors in the modulation of the arbovirus infection of mosquitoes. This concept, known as “immunological cross-talk”, posits that vertebrate blood factors can interact with, and signal through, receptors in the mosquito midgut when imbibed. To date, studies of immunological cross-talk between human blood and vectors has been largely restricted to investigations of *Plasmodium* parasites and Anopheline mosquitoes [24,25,26,27,28,29,30,31]. In particular, the cytokine transforming growth factor β (TGF-β) has been extensively characterized for its ability to regulate innate immune functions in the midguts of Anopheline mosquitoes, with direct consequences for their vector competence for *Plasmodium* parasites. Specifically, low levels of ingested TGF-β directly decrease malaria parasite burdens [25,26,30,31]. Whether TGF-β can exert such effects in *Aedes* spp. mosquitoes and arboviruses is presently unknown.

Thus, in the present study, we investigated whether the hematologic changes caused by T2DM could affect the susceptibility of *Ae. aegypti* mosquitoes to ZIKV. Both artificial bloodmeals and viremic animals were utilized to produce bloodmeals with a “diabetic phenotype”. The artificial bloodmeals were produced utilizing human erythrocytes that underwent in vitro glycosylation to increase the glycosylation of hemoglobin on the cell surface, often used as a diagnostic tool for glycemic control and diabetes, with a HbA1C ≥6.5% being a diagnostic marker of T2DM [32,33]. To expose mosquitoes to viremic diabetic animals, we utilized the leptin-receptor mutant LEPR^DB/DB^ model on the C57BL6 mouse background. Following the consumption of ZIKV via artificial bloodmeals or bloodmeals from viremic animals, the rates of infection and dissemination from the midgut into the hemocoel were calculated following a set extrinsic incubation period. In both the in vitro and in vivo experimental paradigms, we observed significant increases in *Aedes aegypti*’s susceptibility to ZIKV from phenotypically diabetic bloodmeals compared to control bloodmeals.

## 2. Materials and Methods 

### 2.1. Cells and Viruses

African green monkey kidney cells (CCL-81, hereafter referred to as Vero), were purchased from the American Type Culture Collection (ATCC, Bethesda, MD, USA) and maintained in Dulbecco’s Modified Eagle’s Medium (DMEM, ThermoFisher Scientific, Waltham, MA, USA) supplemented with 5% (*v*/*v*) heat-inactivated fetal bovine serum (FBS, Atlanta Biologicals, Flowery Branch, GA, USA), 1% (*v*/*v*) Penicillin–Streptomycin (P/S, ThermoFisher Scientific, Waltham, MA, USA; 100 U/mL and 100 μg/mL, respectively) in a humidified 37 °C incubator with 5% CO_2_. The ZIKV strain PRVABC59 (Puerto Rico, 2015) was received as a lyophilized stock (World Reference Center for Emerging Viruses and Arboviruses, UTMB) as a passage 4 stock. The virus underwent two additional Vero cell passages to generate the stocks utilized in these studies.

### 2.2. Animal Procedures

All the procedures utilizing animals were conducted in full compliance with the guidelines established by the Animal Welfare Act for the housing and care of laboratory animals and conducted as laid out in the University of Texas Medical Branch Institutional Animal Care and Use Committee (UTMB-IACUC)-approved Protocol #1708051A. In experiments conducted with LEPR^DB/DB^, LEPR^WT/DB^ and C57BL/6J mice, 10-week-old animals (*n* = 4 per group per experiment) were pretreated with 1.5–2.0 mg of IFNAR-blockading antibody (MAR1-5A3, Leinco, St. Louis, MO, USA) to render them permissive for ZIKV viremia. A 300 μL maximum dosage was allowed; therefore, the functional dose was contingent on the concentrations provided in the manufacturer vial (concentration range of 5.0 to 7 mg/mL). The dose range of 1.5 mg or 2.0 mg of anti-IFNAR blockade was based on previous reports and manufacturer protocols [34]. Animals underwent antibody treatment one day before and one day after infection with ZIKV. On day 0, mice were infected via the intraperitoneal route with 5 log_10_ FFU of ZIKV in a volume of 100 μL of phosphate buffered saline (PBS). At three or four days after exposure to ZIKV, the mice were anesthetized via intraperitoneal inoculation with ketamine (100 mg/kg) and xylazine (10 mg/kg). Following the anesthesia, the mice were placed atop the mesh lid of a 0.5 L cardboard cup containing sugar-starved *Ae. aegypti*. Mosquitoes were allowed to feed for 30 min and then cold-anesthetized, and fully engorged specimens were incubated as described below. After blood feeding, the mice were humanely euthanized and bled to quantify the viremia by focus-forming assays.

### 2.3. Preparation and Administration of Artificial Bloodmeals

All the artificial bloodmeals were created with a calculated titer of 4.3 log_10_ FFU/mL of ZIKV. Citrated single-donor human whole blood (gender unspecified, Lampire Biological Laboratories, Pipersville, PA, USA) was used as previously described [35], with the following modifications: The first type of bloodmeal was supplemented with 0.5 ng/mL, 5 ng/mL, or 50 ng/mL recombinant transforming growth factor β1 (rTGFβ-1) (R&D Systems, Minneapolis, MN, USA) (to act as a 100-fold range that is reported for human health and disease) [36,37]. The second type of bloodmeal utilized artificially glycosylated human erythrocytes. Briefly, erythrocytes were washed as previously described and resuspended in glycation buffer (45 mmol/L glucose in DPBS) to produce a 10% hematocrit suspension (volume: 30 mL) [38,39], and 300 μL of 100 X P/S was added. These glycation reactions were incubated for 18 h at 37 °C in a shaking incubator, centrifuged (200 RCF, 15 min) and resuspended in a 5 mL residual volume. A 20 μL sample was analyzed utilizing an A1CNow + test kit (PTS Diagnostics, Whitestown, IN, USA) to determine the glycation of the erythrocytes. A control bloodmeal was mock glycated and processed identically, but resuspended and incubated in PBS instead of glycation buffer. The mock-glycosylated blood was compared against unmodified donor whole blood to confirm identical A1C readouts.

### 2.4. Mosquitoes

Adult female *Aedes aegypti* from two colonies, Galveston Texas (F5), or Rockefeller, unknown generation (ROCK), were used in these studies. The mosquitoes were housed prior to and following the ingestion of bloodmeals in an incubator with a temperature of 27 ± 1 °C with 80 ± 10% relative humidity and a 16:8 light:dark cycle. The mosquitoes were housed in 0.5 L cardboard cups with mesh lids and provided ad libitum access to 10% sucrose. For vector-competence analyses, female mosquitoes were sorted 3 days post-eclosion, at which point access to sucrose was removed via replacement with cotton rounds soaked in water, which were removed 4–6 h prior to blood feeding. After feeding, sucrose-soaked rounds were provided once again [35,40,41].

### 2.5. Sampling/Processing Mosquitoes

To determine whether mosquitoes that ingested ZIKV became infected, and whether the infections disseminated from the midgut into the hemocoel, they were anesthetized on ice fourteen days post-bloodmeal, and legs of individual mosquitoes were removed and placed into microfuge tubes containing sterilized steel ball bearings and 500 μL of mosquito collection medium (MCM) (DMEM, 2% FBS, 1% Pen–Strep, and 2.5 μg/mL of amphotericin B). The individual carcasses were placed into separate tubes with 500 μL of MCM and processed by trituration for 5 min at 26 Hz in TissueLyser II (QIAGEN, Hilden Germany), followed by centrifugation at 200× *g* for 5 min [35,40,41,42,43]. 

### 2.6. Detection and Quantification of ZIKV Infection

To determine the presence or absence as well as titers of virus present in the stocks, mouse sera or bloodmeals, samples either underwent 10-fold serial dilutions in dilution medium (DMEM, 2% FBS, and 1% Pen–Strep) in 96-well culture plates as previously described [35,40,41,44,45] or were inoculated directly. Next, 100 μL of samples were added to 80–95% confluent monolayers of Vero cells on either 12- or 24-well tissue culture plates. The viral dilutions were allowed to adsorb for one hour in a humidified 37 °C incubator with 5% CO_2_, and then overlayed using a solution of DMEM containing 3% FBS, 1% Pen–Strep, 1.25 μL/mL amphotericin B, and 0.8% weight/vol methylcellulose. The overlayed plates were incubated for 5 days in a humidified 37 °C incubator with 5% CO_2_; then, each well was washed twice with PBS and fixed for a minimum of 30 min in an ice-cold solution of methanol:acetone, 1:1, vol/vol. Following the fixation, the organic fixative was removed and the plates were air dried. Following complete air drying, the plates were washed with PBS and then blocked with 3% FBS in PBS, followed by an overnight incubation with mouse hyperimmune serum against the ZIKV strain MR-766 (1:2000 in blocking solution) (WRCEVA, UTMB). The plates were then washed with PBS, followed by incubation with a goat anti-mouse secondary antibody conjugated to horseradish peroxidase (KPL, Gaithersburg, MD, USA) diluted 1:2000 in blocking solution. The plates were washed with PBS, after which an aminoethylcarbazole solution (Enzo Diagnostics, Farmingdale, NY, USA) prepared according to the manufacturer′s protocol was added, and the plates were incubated in the dark. Development was halted by washing in tap water, and the plates were allowed to air dry at room temperature before scoring.

### 2.7. Multiplex Cytokine/Chemokine Bead Assay

To determine the levels of cytokines and chemokines in the mice at the time of the bloodmeal, the serum collected after feeding was subjected to two multiplex bead assays: Bio-Plex Pro Mouse Cytokine 23-Plex (Bio-Rad, Hercules, CA, USA). Samples were run in duplicate according to the manufacturer’s instructions, and each value was plotted alongside the average value per animal cohort.

## 3. Results

### 3.1. TGF-β Immunological Cross-Talk in Aedes Aegypti

To establish whether the same types of immunological cross-talk that have been observed in the *Plasmodium*/*Anopheles* system [24,25,26,27,28,29] could potentially occur in the *Ae. aegypti*/ZIKV system, a 100-fold range of rTGF-β1 was added to individual bloodmeals, which were then provided to mosquitoes.

**Figure 1:**
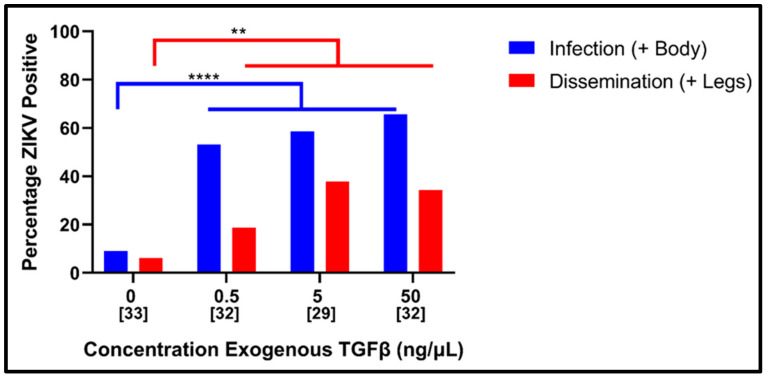
Effect of TGF-β1 on susceptibility of *Ae. aegypti* to ZIKV. Strain PRVABC59 at a titer of 4.2 log_10_ FFU/mL was provided to female mosquitoes (Galveston F5) in artificial bloodmeals that were supplemented with exogenous human TGF-β1 at indicated doses. Blood-fed mosquitoes were incubated at 27 ± 1 °C with 80 ± 10% relative humidity and a 16:8 light:dark cycle for fourteen days, at which point they were collected and analyzed for the presence or absence of ZIKV by Vero cell assays. Data are presented as percentages: 100 * [number of infected bodies (infection) or legs (disseminated infections) over total number of mosquitoes assayed in a given condition]. Numbers in brackets indicate sample sizes. * *p* < 0.05, ** *p* < 0.01, *** *p* < 0.001 and **** *p* < 0.0001.

As demonstrated in Figure 1, the addition of rTGF-β1 to bloodmeals significantly enhanced the percentage of mosquitoes (number positive over number assayed) infected with ZIKV when compared to the control bloodmeal (nominal logistic regression, chi-squared = 29.0, *p* < 0.0001). This analysis showed a significant gain in infection between rTGF-β1 at 0 ng/mL (9.0% of mosquitoes infected) and 0.5 ng/mL (53.1%), with no further significant increases as the rTGF-β1 concentrations increased. A similar pattern was observed for the percentage dissemination (the number with positive legs over total number assayed) (nominal logistic regression, chi-squared = 12.7, *p* < 0.005), although no significant differences among adjacent pairs of concentrations were detected.

### 3.2. Infectivity of ZIKV in Artificially Glycosylated Bloodmeals

To investigate whether the infectivity of ZIKV for *Ae. aeygpti* could be affected by bloodmeals that had markers of T2DM, an in vitro glycosylation strategy was adopted. In this methodology, human erythrocytes were treated with a glycation buffer (45 mmol/L glucose) to increase the level of HbA1c and compared to mock-glycated blood from the same donor. To facilitate comparisons, the HbA1c values for unmanipulated blood were also tested (both the unmanipulated whole blood and mock-glycated blood HbA1c values were found to be 5.5%).

**Figure 2:**
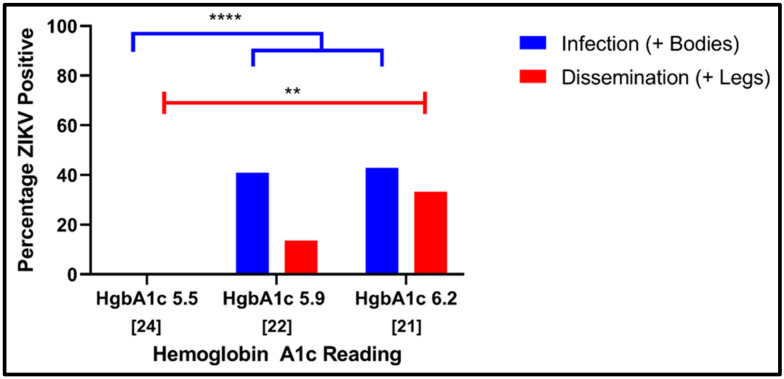
Effect of erythrocyte glycosylation on ZIKV infectivity of mosquitoes. ZIKV strain PRVABC59 was provided to *Ae. aegypti* (Galveston F5) in artificial bloodmeals at titers of 4.1 log_10_ FFU/mL that were made with artificially glycosylated erythrocytes or mock-glycosylated erythrocytes (HbA1c 5.5). Blood-fed mosquitoes were incubated at 27 ± 1 °C with 80 ± 10% relative humidity and a 16:8 light:dark cycle for fourteen days, at which point they were collected and analyzed for the presence or absence of ZIKV. Data are presented as percentages: 100 * [number of infected bodies (infection) or legs (dissemination) over total number of mosquitoes assayed in a given condition]. Numbers in brackets indicate sample sizes. * *p* < 0.05, ** *p* < 0.01, *** *p* < 0.001 and **** *p* < 0.0001.

ZIKV in mock-glycated blood failed to infect any of the tested mosquitoes at a titer of 4.1 log_10_ FFU/m (Figure 2). Strikingly, glycation to HgbA1c levels of 5.9 or 6.2 yielded bloodmeals that infected *Ae. aegypti* at titers of 4.2 (40.9% of the mosquitoes infected) and 4.1 log_10_ FFU/m (42.9%), a significant gain relative to the controls (contingency table analysis with 1 added to every cell to avoid 0 values; chi squared = 24.6, *p* < 0.0001). The disseminated infections also increased with increasing glycation of HgbA1c, but the only significant difference was between the control and the high-glycation bloodmeals (Fisher’s exact test, *p* = 0.007).

### 3.3. LEPR^DB/DB^ Mice Require IFNAR Blockade to Become Viremic

While several mouse models of obesity and diabetes exist, the LEPR^DB/DB^ model was chosen because its obesity and metabolic syndrome do not require researcher manipulation and the mutation that drives the phenotype is not intrinsically immunological in nature. LEPR^DB/DB^ mice are produced on a C57BL/6J background, which failed to become viremic when infected with ZIKV in previous studies [34,46]. To produce and characterize viremia in LEPR^DB/DB^ mice, animals were treated 1 day prior to and 1 day after ZIKV infection with the IFNAR-blockading antibody MAR1-5A3.

**Figure 3:**
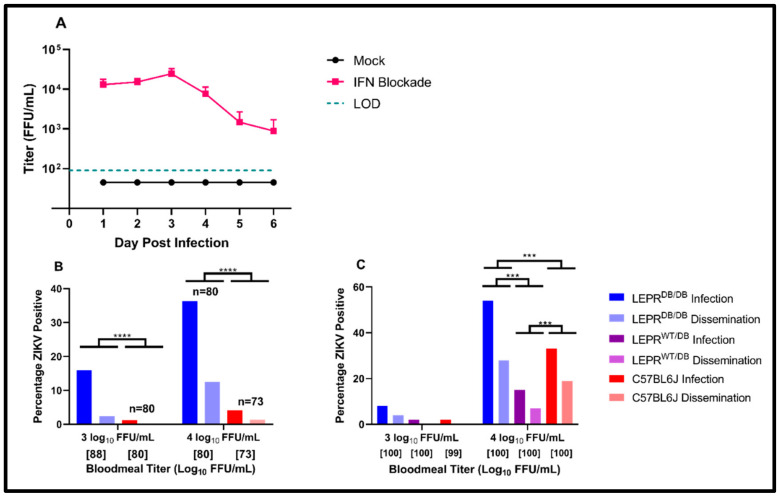
Susceptibility to ZIKV ingested from LEPR^DB/DB^ and control animals. (**A**) LEPR^DB/DB^ were treated with 1.5–1.8 mg of IFNAR-blockading antibody MAR1-5A3 one day prior to and one day after infection with 5.00 log_10_ FFU of ZIKV PRVABC59. Viremia was determined via focus-forming assay performed on serum from retro-orbitally sampled blood. (**B**) ZIKV PRVABC59 was provided to *Ae. aegypti* (Galveston F5) via infected mice (either LEPR^DB/DB^ or C57BL/6J). Mosquitoes were exposed to titers of 4.42 ± 4.02 log_10_ FFU/mL (corresponding to 3 DPI) and 3.56 ± 2.95 log_10_ FFU/mL (4 DPI). Blood-fed mosquitoes were incubated at 27 ± 1 °C with 80 ± 10% relative humidity and a 16:8 light:dark cycle for fourteen days, at which point they were collected and analyzed for the presence or absence of ZIKV via plaque assays. (**C**) ZIKV PRVABC59 was provided to *Ae. aegypti* (Rockefeller) via infected mice (LEPR^DB/^^DB^, LEPR^WT/^^DB^ or C57BL/6J). Mosquitoes were exposed to titers corresponding to 3.68 ± 3.01 log_10_ or 4.32 ± 3.88 log_10_ FFU/mL. Blood-fed mosquitoes were incubated at 27 ± 1 °C with 80 ± 10% relative humidity and a 16:8 light:dark cycle for fourteen days, at which point they were collected and analyzed for the presence or absence of ZIKV via plaque assays. In B) and C), data are presented as percentages: 100 * [number of infected bodies (infection) or legs (dissemination) over total number of mosquitoes assayed]. Numbers in brackets indicate sample sizes. * *p* < 0.05, ** *p* < 0.01, *** *p* < 0.001 and **** *p* < 0.0001.

Concordant with previous experiments in C57BL/6J mice, LEPR^DB/DB^ animals failed to become viremic in the absence of IFNAR blockade. In the animals treated with MAR1-5A3, the viremia peaked three days post-infection at an average titer of 4.4 log_10_ FFU/mL (Figure 3A), with no apparent difference between males and females (data not shown).

### 3.4. Infection of Aedes aegypti with ZIKV PRVABC59 *via* Viremic LEPR^DB/DB^ Mice

Upon establishing that the IFNAR blockade of LEPR^DB/DB^ mice enhanced ZIKV viremia with titers exceeding 4 log_10_ FFU/mL, LEPR^DB/DB^ and C57BL/6J mice were treated with MAR1-5A3 and then presented to *Ae. aegypti* (Galveston F5) either three or four days post-infection. 

Viremic LEPR^DB/DB^ mice were capable of infecting *Ae. aegypti* in the relatively low-titer condition of 3.6 ± 2.9 log_10_ FFU/mL at a rate of 15.9%, with 2.3% disseminated infections (Figure 3B), while at this titer, viremic C57Bl/6J only infected 1.2% of mosquitoes, with no disseminated infections observed. At the higher titer of 4.4 ± 4.0 log_10_ FFU/mL, viremic LEPR^DB/DB^ infected 36.3% of the *Ae. aegypti*, and 12.5% of the mosquitoes demonstrated disseminated infections. At the same virus titer, the infected control animal (C57BL/6J) demonstrated substantially lower rates of infection (4.1%) and disseminated infection (1.4%) in this population of *Ae. aegypti.* A logistic fit of the data revealed that both the mouse strain and virus titer had a significant effect on infection (DF = 1 for each parameteter, *p* < 0.0001 and *p* = 0.002, respectively) as well as dissemination (DF = 1 for each parameter, *p* = 0.0001 and *p* = 0.004, respectively), with higher infection resulting from higher titers and LEPR^DB/DB^ mice. 

To corroborate and extend these findings, *Ae aegypti* ROCK mosquitoes, which are highly permissive to infection, were allowed to feed upon viremic homozygous LEPR^DB/DB^, heterozygous LEPR^WT/DB^ and wild-type C57BL/6J animals with viremia titers of ≈3 or 4 log_10_ FFU/mL.

In this system, minimal infection and dissemination was observed after the ingestion of 3 log_10_ FFU/mL (Figure 3C). At titers about 10-fold higher, the infection and dissemination increased significantly in mosquitoes exposed to all the animals (DF = 1, chi squared = 112.6, *p* < 0.0001). With the homozygous mouse strain LEPR^DB/DB^, infection reached rates of 54% with 28% dissemination. In this strain of mosquitos, however, titers of ≈4 log_10_ FFU/mL in C57BL/6J mice yielded 33% infection and 19% dissemination. Mosquitoes exposed to heterozygous LEPR^WT/DB^ animals with this viremia titer demonstrated the lowest rates, of 15% infection and 7% dissemination. A nominal logistic model revealed significant differences among each pair of genotypes (*p* < 0.001 for each pairwise comparison).

To determine whether serum cytokines were associated with the differences in mouse phenotype and, therefore, infection and disseminated infection, mouse sera were collected immediately after bloodmeals and subjected to multiplex bead analysis for 23 cytokines (Table 1; data for each individual mouse are provided in Supplementary Table S1). To reduce the number of comparisons and the possibility of false discoveries, the cytokines were first grouped into one of four classes (Figure S1) [47]: pro-inflammatory (IL-1 α, IL-1β, IL-6, IL-13, 1L-17A, G-CSF, IFN-g and TNFa), anti-inflammatory (IL-10, IL-12p40 and IL-12p70), adaptive immunity (IL-2, IL-3, IL-4, IL-5, IL-9 and GM-CSF) and chemokines (eotaxin, KC, MCP-1, MIP-1 α, MIP-1 β and RANTES). Each group was subject to principal component (PC) analysis, and for each group, the first PC explained substantially more variation in the data than subsequent PCs. Thus, we tested for the variation in the PC-1 of each group using a Wilcoxon test. Only the chemokines showed a significant difference among genotypes (DF = 2, chi-squared = 6.04, *p* = 0.049); while the wild-type was higher than either the heterozygous or homozygous genotype, a post hoc test revealed a significant difference only between the wild-type LEPR and the heterozygote (*p* = 0.03). The PC-1 of the chemokines was significantly associated with positive values of MCP-1, MIP-1β and KC and, to a lesser degree, negative values of MIP-1α and RANTES. However, we observe that one mouse (number 4; see Supplementary Table S1) was flagged as an outlier in most comparisons. While we chose not to remove this individual, doing so would have changed the significance of the comparison.

## 4. Discussion

To date, there have been limited data on the impacts of T2DM on the arbovirus infection of vector mosquitoes. Herein, we utilized manipulated bloodmeals and mouse models of T2DM to assess the impact of “diabetic” blood on ZIKV infectivity for the major vector, *Ae. aegypti*.

The incorporation of recombinant mammalian TGF-β1 in artificial bloodmeals increased ZIKV infectivity in *Ae. aegypti*. Additionally, ZIKV presented in the context of phenotypically diabetic in vitro and in vivo bloodmeals (artificially glycosylated/viremic LEPR^DB/DB^ mice) was more infectious. The effect of TGF-β on immunological cross-talk in Anopheline mosquitoes and *Plasmodium* parasites has previously been examined [25,26,28,30,31,48]. Specifically, ingesting mammalian TGF-β at low doses can induce *Plasmodium*-parasite killing in *Anopheles stephensi* mosquitoes via the induction of NO synthase (AsNOS) [30]. In vitro and ex vivo experiments conducted with dengue virus-1 (DENV1) have previously demonstrated the sensitivity of flaviviruses to NO in the context of isolated human monocytes [49]. Our data appear to differ from these previous studies, as the rate of ZIKV infection and dissemination was higher in bloodmeals with higher TGF-β1, regardless of the dosage. This pattern suggests that TGF-β1 does not have a similar effect on AsNOS in *Ae. aegypti* mosquitoes to in Anophelines, or that TGF-β1 stimulates other pathways that favor ZIKV infection and thereby counteract the negative impacts of NO, and/or that ZIKV is less sensitive to NO than DENV. Therefore, a mechanistic examination of how mammalian TGF-β1 affects Aedes mosquitoes and ZIKV infection in vivo is needed.

The examination of the role of HgbA1c in the ZIKV infection of *Ae. aegypti* revealed significantly greater infection in the presence of elevated glycosylation. Blood from a single donor was subjected to artificial glycosylation [38,39], and the resultant erythrocytes were used to prepare in vitro bloodmeals. While a promising preliminary analysis, this methodology is highly artificial. Follow-up analyses would ideally be conducted using whole blood from donors representing a wider range across the HgbA1c spectrum (healthy, prediabetes and diabetes). The disadvantage of such an approach is the intrinsic variability in various hematologic parameters among blood donors. Of particular note, the role of blood glucose is one that is critically important in future analyses. In the latter phases of T2DM, after β-cell dysfunction and death, plasma insulin falls and plasma glucose rises [50,51,52,53], underscoring the value of using animal models that can exhibit a dynamic range of both insulin and glucose. One recent analysis demonstrated that mosquitoes imbibing a bloodmeal supplemented with glucose had significantly increased DENV titers at both 3 and 7 days after an infectious bloodmeal [54]. This was also accompanied by an increase in DENV envelope protein levels as a function of the concentration of glucose and days post-infectious bloodmeal [54]. However, greater concentrations of glucose have also been shown to inhibit in vitro ZIKV replication in human kidney cells [55], indicating the potential for acutely hyperglycemic patients to have decreased viremia when infected with ZIKV. These analyses reinforce the value of in vivo analyses that examine the role of glucose and insulin in the flavivirus infection of vector mosquitoes.

We also used mouse models of T2DM to investigate the impacts of this condition on ZIVK infection for mosquitoes. ZIKV in the blood of LEPR^DB/DB^ mice infected Galveston *Ae. aegypti* more efficiently than virus from the non-obese genetic background, C57BL/6J. When the highly susceptible Rockefeller strain of *Ae. aegypti* was tested using LEPR^DB/DB^, LEPR^WT/DB^ or control mice with a high ZIKV viremia, the DB homozygous mice infected a significantly higher proportion than the heterozygotes. These results must be considered in light of other recent studies probing the impact of mammalian insulin on mosquito immunity and susceptibility to arboviruses. Ahlers et al. showed that mammalian insulin can trigger Akt and ERK signaling in mosquitoes, leading to the transcription of JAK/STAT-associated antiviral genes [56]. Moreover, the pre-treatment of cells from both *Culex* and *Aedes* mosquitoes with mammalian insulin suppressed the replication of West Nile virus, a finding largely recapitulated in *Ae. albopictus* C6/36 cells with both DENV and ZIKV [56].

We utilized phenotypically T2DM animals, and the serum insulin was not quantified. Nonetheless, it is well documented that the LEPR^DB/DB^ model becomes hyperinsulinemic as early as 10–14 days of age, with the peak levels of insulin observed at 3 months of age, and a drop off in insulin precipitated by β-cell dysfunction and depletion by 6 months of age occurs [57]. In contrast, LEPR^WT/DB^ animals do not become obese or even hyperinsulinemic at any age [50,58]. The mice we used were approximately 2.5 months old, and therefore, the DB homozygotes were in a hyperinsulinemic state, whereas the heterozygotes were not. At first consideration, the finding that the DB homozygotes infected significantly more *Ae. aegypti* with ZIKV than the heterozygotes seems to contradict the finding that insulin signaling decreases WNV replication in *Culex* mosquitos [56]. However, it is possible that the extremely high levels of insulin produced by the DB homozygotes disrupted rather than enhanced JAK/STAT signaling. Further studies utilizing transcriptomics to investigate this possibility are warranted. It is worth noting that the LEPR^DB/DB^ mice demonstrate serum insulin levels of ≈ 1–5 ng/mL, corresponding to 2.25 to 11.23 PM [50,59]. These are levels 15.2- to 75.6-fold lower than the 17 PM utilized to produce the phenotype observed in *Culex* mosquitoes exposed to WNV [56]. This is particularly important in that, in the current study, mosquitoes took bloodmeals from viremic animals rather than from an artificial feeder spiked with non-physiologic levels of insulin, potentially convoluting the infection outcomes. Regarding the discussion of blood glucose above and blood lipids below, it will also be important to completely characterize the blood composition in the DB homozygous and heterozygous mice in future experiments. Finally, the IFNAR blockade may have obscured or even altered the impact of insulin on JAK–STAT signaling. Unfortunately, most small animal models of ZIKV rely on immunological defects such as non-functional interferon α/β receptors, to allow the virus to productively infect [46,60]. The ablation of mouse STAT2 (which is known to resist ZIKV antagonism) and its replacement with human STAT2 have been demonstrated to enhance ZIKV replication in immunocompetent (C57BL/6N) mice [61], raising the possibility of rendering such animals obese via dietary supplementation for subsequent experiments.

Among a panel of cytokines measured, only the chemokines, particularly MCP-1, MIP-1β and KC, differed among the three mouse genotypes, with the levels in heterozygote mice lower than those in both LEPR^DB/DB^ and wild type mice, albeit the difference was only significant for the heterozygote–wild-type comparison. We have no immediate explanation for this variation, and it may reflect a complex interaction between the effects of the mouse genotype *per se*, ZIKV infection and exposure to mosquito saliva on chemokines. It is intriguing that the heterozygote mice also infected a smaller percentage of mosquitoes than either the LEPR^DB/DB^ or wild-type mice. Koerber-Rosso et al. conducted a review of studies comparing non-human organisms that carried mono-allelic, likely pathogenic, variants at the LEPR locus (hereafter heterozygotes) to their wild-type counterparts [62]. These studies showed considerable variation in the effects of the heterozygous state on body weight and metabolic state. Moreover, to our knowledge, there are relatively few published studies comparing cytokines in mice heterozygous at the LEPR allele to wild-type mice [63]. Thus, further studies are needed to characterize the cytokine responses of these heterozygotes to infection.

Our study has several limitations, including the fact that the methodology utilized to artificially glycosylate erythrocytes failed to produce glycosylation levels equivalent to those observed in clinical diabetes; the highest HbA1c value we achieved was 6.2%, which fails to meet the 6.5% cutoff for a diagnosis of T2DM [32,33]. From an experimental point of view, artificial glycosylation allows for the erythrocytes of the same donor to be used as both the test article and the control condition and eliminates the confounding variable of using multiple donors. However, future studies using human blood from T2DM and control individuals are needed. Another limitation is that the LEPR^DB/DB^ hyperlipidemia mouse model does not entirely recapitulate human T2DM and obesity. Human T2DM is generally characterized by high LDL and VLDL, and low HDL cholesterols, while LEPR^DB/DB^ animals have highly elevated levels of HDL. Previous studies have demonstrated that host cholesterols are critical for the flavivirus infection of both mosquitoes and mammals (reviewed in [64]). In at least one analysis, the administration of LDL to mosquito cell lines (Aag2 and C6/36) decreased ZIKV infectivity, and the supplementation of an artificial bloodmeal with 50 mg/dL of LDL reduced the levels of ZIKV RNA compared to that in mosquitoes fed control bloodmeals. Conversely, however, the treatment of Aag2 and C6/36 cells with HDL failed to affect ZIKV replication [65]. These results suggest that our findings may be specific to the LEPR^DB/DB^ model, necessitating corroboration in alternative models of diabetes, such as via high-fat diets in wild-type animals [66,67].

Given the increasing propensity for arboviral and zoonotic agents to emerge due to climate change, deforestation and the expansion of the human population into areas associated with enzootic circulation [10,68,69], as well as the increase in chronic conditions in the human population [7,8,21,70], it is of paramount importance that vector competence studies incorporate mammalian factors influenced by chronic conditions. Due to the common finding that pre-existing medical conditions alter the pathogenesis of arboviral pathogens [23], individuals with such conditions are, at the very least, a vulnerable population to consider epidemiologically when striving to control outbreaks. Furthermore, our data suggest that that these populations represent a hitherto-unconsidered driver of arbovirus transmission.

## Figures and Tables

**Table 1:** Cytokine levels in mice varying in the LEPR mutation and infected with ZIKV. Serum was taken from animals immediately following *Aedes aegypti* Rockefeller feeding. Serum from each animal was run in duplicate utilizing a mouse multiplex bead assay to determine circulating levels of 23 cytokines. Data are presented as averages of all animals of a given phenotype, alongside standard deviations. LOD: limit of detection; N/A: not applicable.

	C57BL/6J		LEPR^WT/DB^		LEPR^DB/DB^	
Cytokine	Mean (pg/mL)	Standard Deviation (pg/mL)	Mean (pg/mL)	Standard Deviation (pg/mL)	Mean (pg/mL)	Standard Deviation (pg/mL)
**IL-1α**	14.82	16.12	4.45	1.57	2.85	2.86
**IL-1β**	<LOD	N/A	<LOD	N/A	<LOD	N/A
**IL-2**	2.89	2.61	1.41	1.45	1.13	0.47
**IL-3**	3.76	2.36	5.36	1.16	3.58	1.39
**IL-4**	0.51	0.66	0.31	0.28	<LOD	N/A
**IL-5**	0.92	0.58	0.75	0.69	<LOD	N/A
**IL-6**	14.46	7.30	9.74	4.63	6.73	3.14
**IL-9**	13.31	6.97	15.96	2.85	13.85	2.09
**IL-10**	10.60	5.29	11.98	3.67	8.30	3.79
**IL-12 (p40)**	2158.22	1456.44	1871.29	1452.92	1387.81	754.73
**IL-12 (p70)**	47.80	32.93	55.44	5.58	48.39	9.13
**IL-13**	62.10	47.67	44.25	4.58	51.31	8.05
**IL-17A**	41.86	26.50	52.88	34.07	59.14	24.63
**Eotaxin**	2548.90	808.52	2747.01	458.67	3717.02	411.93
**G-CSF**	337.84	74.60	164.98	73.54	159.89	101.84
**GM-CSF**	5.96	6.29	<LOD	N/A	6.25	4.36
**IFN-γ**	86.00	141.91	14.37	3.81	17.52	8.78
**KC**	138.36	53.52	57.20	15.66	102.53	30.01
**MCP-1**	1068.20	94.62	542.34	105.48	826.28	298.69
**MIP-1α**	3.55	1.21	3.72	1.04	4.94	0.94
**MIP-1β**	714.95	1057.70	168.44	19.14	217.29	31.72
**RANTES**	452.30	216.71	458.00	239.11	472.09	26.57
**TNF-α**	47.40	14.87	167.51	189.88	48.67	10.34

## Data Availability

Data is contained within the article or Supplementary Materials. Additionally, raw data are available from the authors upon request.

## Supplementary Materials

The following supporting information can be downloaded at: https://www.mdpi.com/article/10.3390/v14040665/s1. Table S1. Serum was taken from animals at the time point corresponding to the time *Aedes aegypti* Rockefeller fed upon the animals. Serum from each animal was run in duplicate utilizing a mouse multiplex bead assay to determine circulating levels of 23 cytokines at time of feeding. Data shown for each individual animal. Figure S1. Cytokine outputs from Bioplex analysis were categorized as (IL-1 α, IL-1β, IL-6, IL-13, 1L-17A, G-CSF, IFN-g, TNFa), anti-inflammatory (IL-10, IL-12p40, IL-12p70), adaptive immunity (IL-2, IL-3, IL-4, IL-5, IL-9 GM-CSF) or chem-okines (eotaxin, KC, MCP-1, MIP-1 α, MIP-1 β, RANTES). PC-1 analyses for each category is demonstrated in relation to mouse genotype. (A) proinflammatory, (B) chemokines, (C) anti-inflammatory, and (D) adaptive immunity.
