# Supplementary material for: Aedes aegypti Shows Increased Susceptibility to Zika Virus via Both In Vitro and In Vivo Models of Type II Diabetes
Source: Viruses. 2022 Mar 23;14(4):665. doi: 10.3390/v14040665 (PMC9024453; doi:10.3390/v14040665)
Supplement: Supplementary file 1 [file viruses-14-00665-s001.zip › viruses-1591434-supplementary.pdf]

Supplemental Table S1. Serum was taken from animals at the time point corresponding to the time *Aedes aegypti* Rockefeller fed upon the animals. Serum from each animal was run in duplicate utilizing a mouse multiplex bead assay to determine circulating levels of 23 cytokine at time of feeding. Data shown for each individual animal.

	**Wild Type (C57BL6)**				**LEPR$^{WT/DB}$ HETEROYZYGOUS**				**LEPR$^{DB/DB}$ OBESE**			
	Mouse 1	Mouse 2	Mouse 3	Mouse 4	Mouse 5	Mouse 6	Mouse 7	Mouse 8	Mouse 9	Mouse 10	Mouse 11	Mouse 12
**IL-1α**	6.33	10.47	3.84	38.65	4.31	2.74	6.54	4.19	6.76	3.11	1.26	0.28
**IL-1β**	<LLOD	<LLOD	<LLOD	<LLOD	<LLOD	<LLOD	<LLOD	<LLOD	<LLOD	<LLOD	<LLOD	LLOD
**IL-2**	0.95	2.57	1.37	6.66	0.29	0.74	3.53	1.06	1.58	1.47	0.85	0.62
**IL-3**	0.35	4.39	4.53	5.77	5.5	4.94	6.88	4.12	4.12	3.57	1.67	4.94
**IL-4**	0.2	0.18	0.18	1.5	0.18	0.18	0.73	0.18	<LLOD	<LLOD	<LLOD	LLOD
**IL-5**	0.45	1.11	0.45	1.66	0.31	1.77	0.45	0.45	<LLOD	<LLOD	<LLOD	LLOD
**IL-6**	15.42	20.73	4.01	17.66	7.92	6.12	16.52	8.38	9.85	7.41	7.31	2.36
**IL-9**	3.54	16.5	13.57	19.63	13.27	19.91	14.75	15.92	11.76	14.46	12.67	16.5
**IL-10**	3.01	11.09	13.46	14.85	7.12	13.46	15.77	11.57	3.93	7.12	12.99	9.14
**IL-12p40**	953.74	849.51	3269.72	3559.91	2120.43	825.99	3828.41	710.32	2119.06	851.68	1948.91	631.6
**IL-12p70**	1.02	61.68	51.4	77.11	51.89	54.35	51.89	63.62	37.43	47.45	48.93	59.73
**IL-13**	50.21	25.56	40.65	131.96	40.65	40.65	45.47	50.21	54.88	40.65	59.48	50.21
**IL-17A**	71.56	47.48	41.09	7.32	49.25	32.64	102.06	27.56	74.08	27.62	82.69	52.17
**Eotaxin**	1805.65	2754.86	2035.89	3599.19	2688.87	2599.6	2308.99	3390.57	3504.48	4242.21	3826.26	3296.11
**G-CSF**	317.25	331.13	262.45	440.52	126.34	147.36	273.22	112.98	241.21	227.44	152.01	18.9
**GM-CSF**	2.81	2.81	2.81	15.39	<LLOD	<LLOD	<LLOD	<LLOD	7.47	2.81	11.89	2.81
**IFN-γ**	18.19	14.16	12.81	298.84	12.81	12.81	11.8	20.04	11.97	30.62	14.16	13.32
**KC**	182.1	65.64	130.79	174.91	50.33	47.03	80.55	50.88	128.81	121.03	98.38	61.9
**MCP-1**	1110.35	996.5	984.21	1181.73	691.47	447.12	497.78	533	1164.54	754.66	930.17	455.74
**MIP-1α**	3.57	3.1	5.2	2.33	2.45	3.33	4.37	4.74	4.35	5.14	6.17	4.08
**MIP-1β**	189.8	174.99	193.57	2301.45	148.22	168.32	163.08	194.15	204.15	238.43	247.68	178.89
**RANTES**	309.46	445.59	760.81	293.34	377.41	347.02	812.98	294.59	492.23	471.38	489.97	434.77
**TNF-α**	59.74	29.85	40.26	59.74	42.01	152.29	440.7	35.03	43.77	36.77	59.74	54.38

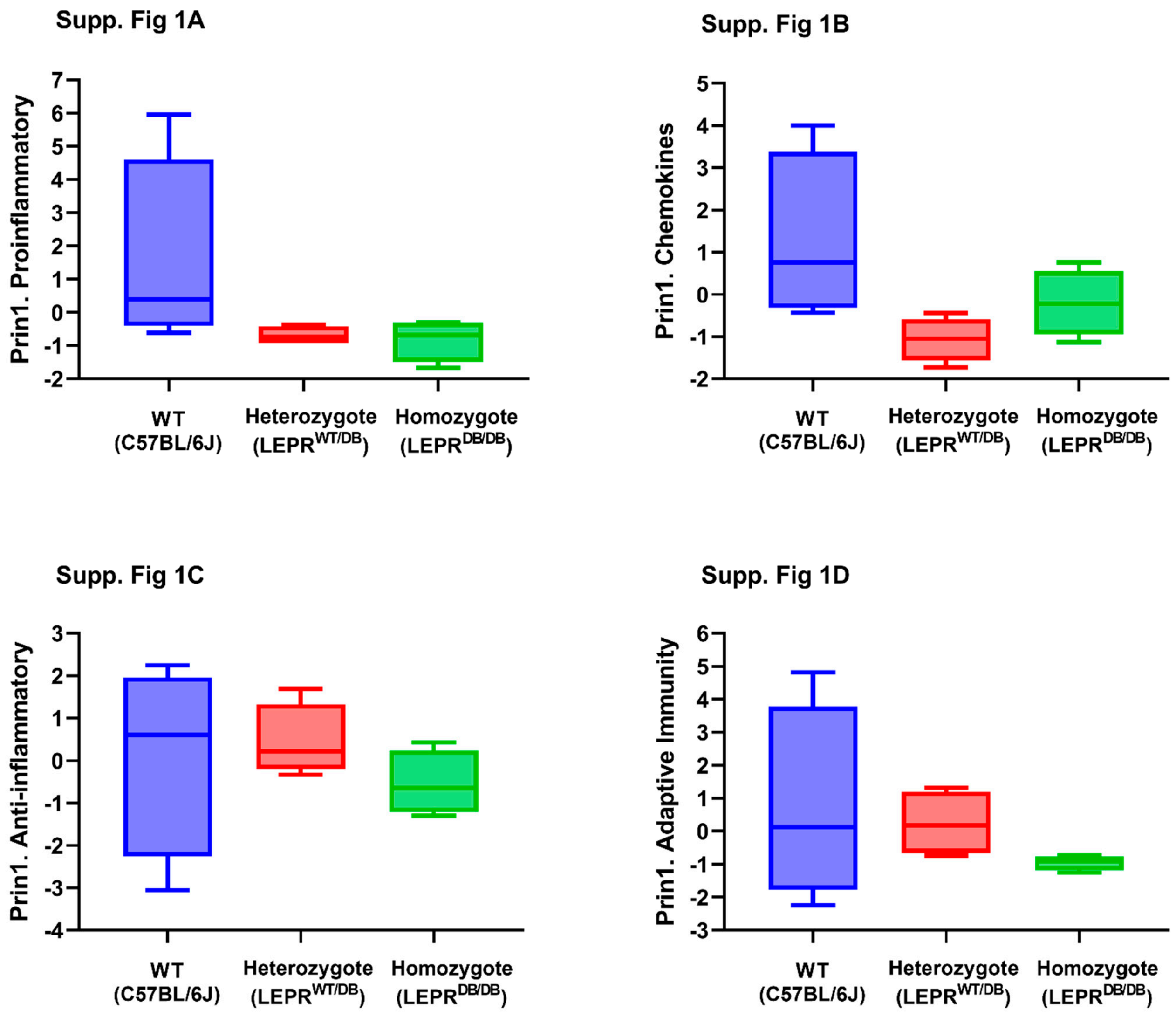

Supplemental Figure S1. Cytokine outputs from Bioplex analysis were categorized as (IL-1 α, IL-1β, IL-6, IL-13, 1L-17A, G-CSF, IFN-g, TNFa), anti-inflammatory (IL-10, IL-12p40, IL-12p70), adaptive immunity (IL-2, IL-3, IL-4, IL-5, IL-9 GM-CSF) or chem-okines (eotaxin, KC, MCP-1, MIP-1 α, MIP-1 β, RANTES). PC-1 analyses for each category is demonstrated in relation to mouse genotype. A) proinflammatory, B) chemokines, C) anti-inflammatory, and D) adaptive immunity.
